# Epidemic-associated Neisseria meningitidis detected by multilocus enzyme electrophoresis.

**DOI:** 10.3201/eid0102.950203

**Published:** 1995

**Authors:** M W Reeves, B A Perkins, J D Wenger

**Affiliations:** National Center for Infectious Diseases, Centers for Disease Control and Prevention (CDC), Atlanta, Georgia, USA.


### Epidemic-Associated *Neisseria meningitidis* Detected by Multilocus Enzyme Electrophoresis

In Oregon and parts of Washington State, the incidence of serogroup B meningococcal disease increased substantially in 1994 (1). Multilocus enzyme electrophoresis (MEE) subtyping of *N. meningitidis* serogroup B strains collected in these areas during 1993 and 1994 suggested that these increases were due to a group of genetically related strains of the enzyme type-5 (ET-5) complex. ET-5 *N. meningitidis* serogroup B were first recognized in Norway in 1974 as the cause of a meningococcal disease epidemic that persisted through 1991. Since 1974, serogroup B meningococci of the ET-5 complex have caused epidemics in Europe, Cuba, and South America; these epidemics elevated disease rates for many years in the affected areas (2,3) and led to sustained efforts for vaccine development. This report describes the use of MEE to compare invasive *N. meningitidis* serogroup B meningococcal strains from Oregon and Washington with epidemic serogroup B strains from other countries and with serogroup B strains that have caused endemic disease in other parts of the United States.

MEE, first described in 1966 as a molecular approach to the study of genetic variation in eukaryotic systems, has only gradually been adopted by microbiologists and epidemiologists. The fundamental concept underlying MEE is that differences in the electrophoretic mobility of constitutive enzymes (resulting from amino acid substitutions) reflect the chromosomal genotype of strains and thereby allow the calculation of a genetic-relatedness index (Figure 1). As recently as 1984, only one bacterial species, *Escherichia coli*, had been studied by MEE. Since then, however, MEE has been used to characterize genetic variation among populations of *Legionella* spp., *Bordetella* spp., *Haemophilus influenzae*, *Streptococcus* spp., *Listeria monocytogenes*, *Neisseria meningitidis*, and other bacteria (4).

To carry out MEE, crude aqueous extracts of bacteria are electrophoresed in a block of 11% to 12% starch in the presence of a dilute buffer (pH 8.0). The block is then cut into thin slices, which are stained to detect specific enzymes. The distance traveled by each enzyme is used to create a series of numbers representing the set of enzyme mobilities characteristic of individual strains. The number of enzymes used is somewhat arbitrary and varies between organisms; 15 to 24 enzymes have usually been adequate to characterize genetic diversity among bacterial populations. For this investigation, electrophoretic variations in 24 enzymes were used to describe genetic variability among isolates of *N. meningitidis* serogroup B. *N. meningitidis* strains used for this analysis were collected from Oregon (1993-1994, n = 64) and part of Washington State (1993-1994, n = 17; 1992, n = 2; 1990, n = 1; unknown, n = 2); serogroup B meningococcal epidemics outside the United States (1976-1993; Norway n = 1; Cuba n = 1; Brazil n = 1; and Chile n = 2); and active population-based surveillance for meningococcal disease in selected areas of the United States (1991-1994, from the San Francisco Bay area, Georgia, Maryland, Oklahoma, and Tennessee, n = 57). The epidemic strains tested from Norway and Cuba are the type used for the outer membrane protein vaccines developed and tested in these countries.

The MEE data analyzed here (Figure 1) suggest that the increased rates of disease in Oregon and part of Washington are caused by highly genetically related *N. meningitidis* serogroup B strains of the ET-5 complex. These strains have been relatively rare in the United States. Oregon and Washington strains match a strain isolated in Santiago, Chile, during 1993. The prolonged duration of some ET-5 serogroup B meningococcal epidemics in large regions (e.g., Brazil, Argentina, and Chile) demands careful monitoring of this organism in the United States. Efforts to identify potentially modifiable risk factors for the disease and develop a vaccine have been intensified. MEE will continue to be the primary means for epidemiologic tracking and surveillance of ET-5 complex *N. meningitidis* serogroup B in the United States.

**Michael W. Reeves,* Bradley A. Perkins***
**Marion Diermayer,[†] and Jay D. Wenger***
*National Center for Infectious Diseases, Centers for Disease Control and Prevention (CDC), Atlanta, Georgia, USA
[†]Emerging Infections Program, Oregon Dept. of Health, Portland, Oregon, USA, and Epidemiology Program Office, CDC, Atlanta, Georgia, USA

### References

1. Centers for Disease Control and Prevention. Serogroup B meningococcal disease—Oregon, 1994. MMWR 1995;44:121-4.
2. Caugant DA, Froholm LO, Bovre K, Holten E, Frasch CE, Mocca LF, Zollinger WD, Selander RK. Intercontinental spread of a genetically distinctive complex of clones of *Neisseria meningitidis* causing epidemic disease. Proc Natl Acad Sci USA 1986;83:4927-31.
3. Sacchi CT, Pessoa LL, Ramos SR, Milagres LG, Camargo MCC, Hidalgo NTR, Melles CEA, Caugant DA, Frasch CE. Ongoing group B *Neisseria meningitidis* epidemic in São Paulo, Brazil, due to increased prevalence of a single clone of the ET-5 complex. J Clin Microbiol 1992;30:1734-8.
4. Selander RK, Caugant DA, Ochman H, Musser JM, Gilmour MN, Whittam TS. Methods of multilocus enzyme electrophoresis for bacterial population genetics and systematics. Appl Environ Microbiol 1986;51:873-84.

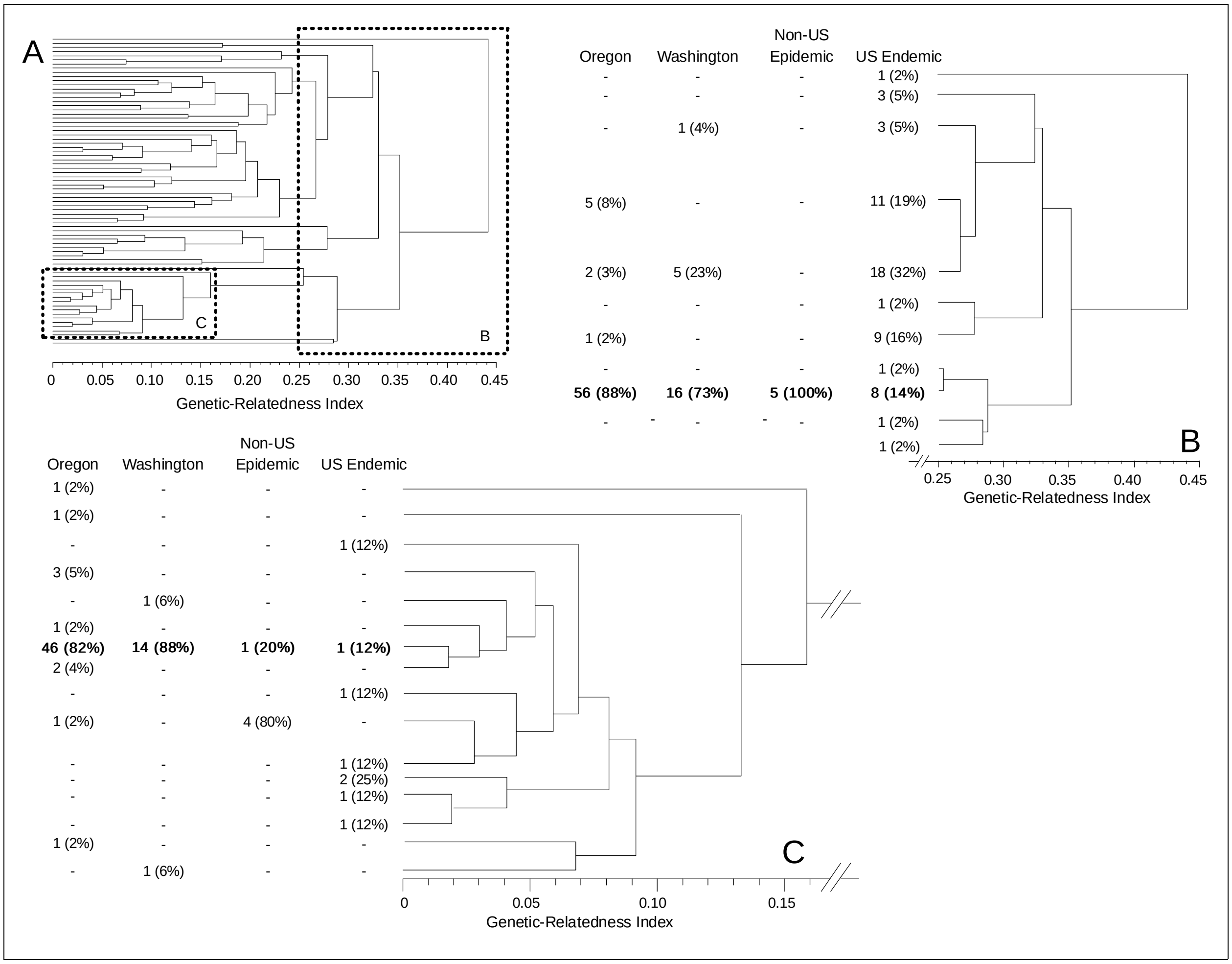

Figure 1. Genetic relatedness of serogroup B strains of *Neisseria meningitidis* from Oregon, Washington, other countries, and endemic-disease cases in the United States.

**A**. *Computer-generated dendrogram for all isolates*; 68 enzyme types (ETs) were identified with the 24 enzymes used in this study. To determine the relatedness of two ETs, start at the left side of the dendrogram at the line (or leg) representing the ET of interest and follow the leg horizontally to the right angle turn (up or down), allowing a path to the other ET. The point on the x-axis at which a right angle turn (up or down) is made to move horizontally back to the left indicates the genetic relatedness. For example, the ET at the top of the dendrogram is related to the other ETs at slightly more than 0.44; the next two ETs are related to each other at an index of approximately 0.17.

**B**. *Expanded view of dendrogram with a genetic-relatedness index of 0.25 to 0.45.* The ET-5 complex cluster is shown in bold type. This portion of the dendrogram represents the population structure of group B meningococci in this study; all strains with a genetic-relatedness level of 0.25 or less are shown as single legs or "complexes" of related strains. The distribution of serogroup B meningococcal strains by site and epidemiologic type (Oregon, Washington, non-U.S. epidemic, and U.S. endemic) and by ET group (or complex) is shown in columns to the left of the dendrogram. At a genetic-relatedness level of 0.25, the dendrogram is divided into 11 ET complexes. The ET-5 complex is the ninth leg down (or third from the bottom), shown in bold type. Of strains endemic in the United States, the highest proportion, 18 (32%) of 56, comprise an ET complex located at the fifth leg from the top and are related to the ET-5 complex at a genetic-relatedness index of just over 0.35. In contrast, 56 (88%) of 64 Oregon strains, 16 (73%) of 22 Washington strains, and 5 of 5 non-U.S. epidemic strains are in the ET-5 complex. Only 8 (14%) of 57 strains endemic in the United States are in the ET-5 complex.

**C.** *Expanded view of ET-5 portion of the dendrogram with genetic-relatedness index of 0 to 0.16.* The distribution of strains by site and epidemiologic type, within the ET-5 complex, is shown to the left of the dendrogram; 16 ETs are represented in the ET-5 complex. The eight strains endemic in the United States in the ET-5 complex are distributed among seven ETs. Forty-six (82%) of 56 Oregon strains and 14 (88%) of 16 Washington strains are clustered at the seventh leg down. One of the five non-U.S. epidemic strains, one of the two strains from Chile, and one (of 56) of strains endemic in the United States match these strains. The other four non-U.S. epidemic strains are located at the tenth leg down, along with one strain from Oregon; these are related to the cluster at the seventh leg at a genetic-relatedness index of approximately 0.06. This slight difference in relatedness results from a difference in the electrophoretic mobility of a single enzyme.

Bacteria strains were provided by K. Steingart, Southwest Washington Health Dist., and M. Goldoft, Washington Dept. of Health.
